# A Biodegradable, Porous Flier Inspired by a Parachute‐Like *Tragopogon* Fruit for Environmental Preservation

**DOI:** 10.1002/smll.202403582

**Published:** 2024-09-17

**Authors:** Stefano Mariani, Kliton Cikalleshi, Marilena Ronzan, Carlo Filippeschi, Giovanna Adele Naselli, Barbara Mazzolai

**Affiliations:** ^1^ Bioinspired Soft Robotics Laboratory Istituto Italiano di Tecnologia Via Morego 30 Genova 16163 Italy; ^2^ The Biorobotics Institute Scuola Superiore Sant'Anna Pontedera 56025 Italy

**Keywords:** aerial seeding, biodegradable flier, colorimetric sensors and indicators, environmental monitoring, parachute and plumed fruit, porous materials, seed dispersal

## Abstract

New devices inspired by flying seeds, or more technically by fruits with dispersal units, could have a significant impact for environmental monitoring and aerial seeding. Among the various types of dispersal units (e.g., winged, gliding), parachuted or plumed units offer the lowest vertical descent speed (i.e., 0.3–0.7 m s^−1^), making them an appealing solution for wind‐driven distribution over large areas. Here, a biodegradable and porous parachute flier based on cellulose acetate, inspired by a *Tragopogon pratensis* fruit is presented. A micrometric‐thick pappus is 3D printed and integrated with a porous colorimetric indicator or a porous beak, with micrometric pores, fabricated through injection molding and leaching techniques. Thanks to the bioinspired design and the lightweight porous structure, the artificial *Tragopogon* mimics the aerodynamics and descent speed of the natural species. Its feasibility is demonstrated in aerial seeding by integrating the beak with a mustard seed (as a model), and in environmental monitoring by coupling it with colorimetric indicators for rain pH and nitrate levels in soils. The proposed flier represents a significant advancement as it is the first parachute‐like biodegradable solution, seamlessly integrated into natural ecosystems, thus contributing to moving a step forward in artificial solutions with zero impact.

## Graphical abstract

A fruit‐like, biodegradable, and porous parachute flier is realized for environmental preservation. The coupling of biological analysis, modeling, design, and additive manufacturing of porous cellulose acetate (with micrometric features) allow the fabrication of a flier resembling the *Tragopogon pratensis* aerodynamics. The fliers offer a solution for colorimetric monitoring of soil pH and nitrate or as carrier for aerials seeding.

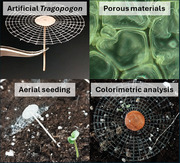

## 1 Introduction

In the framework of environmental preservation, the use of self‐deployable and seed‐inspired systems, or more technically inspired by fruits with dispersal units or diaspores,^[^ ^1^ ^]^ is gaining much interest lately, especially for aerial seeding and monitoring with unmanned aerial vehicle (UAV).^[^ ^2^, ^3^, ^4^, ^5^, ^6^, ^7^, ^8^, ^9^, ^10^, ^11^ ^]^ Frequently, the bioinspiration comes from self‐burying or flying fruits which have evolved their structures to exploit environmental agents, like water/humidity or wind, respectively, to travel far from the parent tree and germinate.^[^ ^12^ ^]^ For example, self‐burying fruits and seeds (e.g., Geraniaceae and Avena) explore soil and self‐bury thanks to the expansion/shrinking of their hygroscopic awns in response to external humidity variations.^[^ ^13^, ^14^ ^]^ On the other hand, flying fruits and seeds rely on their lightweight and drag generating structures to extend their descent time and increase the chances of dispersal by the wind.^[^ ^15^, ^16^ ^]^ Some flying seeds have developed wings for autorotation (e.g., *Tristellateia* and *Acer* ^[^ ^2^, ^16^ ^]^ or for gliding (e.g., *Alsomitra* ^[^ ^17^ ^]^), other exhibit micrometric hairy bristles acting as a parachute (e.g., Dandelion and *Tragopogon* ^[^ ^15^, ^18^ ^]^).

Recently, bioinspired self‐burying artificial systems have been developed for aerial seeding purposes. Luo et al. showed a three‐tailed and self‐drilling carrier inspired by Geraniaceae fruits and seeds (i.e., *Erodium*) made by turning delignified wood veneer. The hygromorphic awn was coupled with a biodegradable capsule made of flour and carried with radish seeds as germination model for aerials seeding purposes driven by humidity variations.^[^ ^8^ ^]^ Fiorello et al. demonstrated the use of a biohybrid self‐dispersing miniature machine using wild oat fruit awns coupled with a biodegradable capsule made of flour and carried with tomato seeds for perspective reforestation and precision agriculture purposes.^[^ ^9^ ^]^

Yet, even if these seed carriers can move on the ground by means of humidity cycles, their travel and autonomous distribution is quite limited in space and time. To achieve larger distances in dispersion from the release point, carriers inspired by flying plant seeds and fruits should be envisioned.

Bioinspired artificial fliers have been developed and conceived, but only for environmental monitoring of physical or chemical parameters.^[^ ^2^, ^3^, ^4^, ^5^, ^6^ ^]^ Roger's group reported the first biodegradable flier, bioinspired by natural *Tristellateia australasiae* fruits and seeds, for colorimetric sensing of environmental parameters, such as pH, heavy metal concentrations, and ultraviolet (UV) exposure.^[^ ^4^ ^]^ More recently, the same group also reported a biodegradable hybrid passive flier (inspired by dandelion and maple), for the colorimetric monitoring of pH, UV exposure, temperature, and chemiresistor‐type gas sensor (NO_2_ and NH_3_).^[^ ^7^ ^]^ Cikalleshi et al., reported a biocompatible artificial *Acer campestre* 3D printed with PLA and photoluminescent particles were sensitive to environmental temperature.^[^ ^6^ ^]^

Here, we propose a porous and lightweight biodegradable flier, inspired by parachute‐like *Tragopogon pratensis* fruit, for both aerial seeding and colorimetric monitoring of soil parameters.

A *Tragopogon* fruit is composed of a large pappus (radius 15–30 mm), made of hairy bristles that unfold radially and are connected in the center to a beak through the ribs.^[^ ^18^ ^]^ It has a parachute vertical type of falling, and its descent speed is slower (0.3–0.7 m s^−1^)^[^ ^18^ ^]^ than other winged dispersal units (e.g., Acer ≈1 m s^−1^).^[^ ^6^ ^]^ The large area of the pappus in an artificial flier could allow the integration of sensors/indicators with relatively large dimensions (>1 cm^2^), with consequent positive effects on the autonomous perspective detection and reading with unmanned aerial vehicles (UAV) cameras.

To the best of our knowledge, there is only one example of *Tragopogon*‐inspired flier for environmental monitoring. It was fabricated in polyimide films through laser cutting and integrated with battery‐free wireless electronic devices for temperature, humidity, light, pressure, magnetic fields, and acceleration monitoring.^[^ ^3^ ^]^ Albeit pioneering, the reported flier is not biodegradable and relies on electronics, which are often costly and generate e‐waste.^[^ ^19^ ^]^

We employ a bioinspired and additive manufacturing approach for the fabrication of deployable artificial *Tragopogon*. In the first step, we studied the morphology, structure, and aerodynamics parameters of the natural *Tragopogon pratensis* seeds for identifying key principles relevant to the design, modeling, and development of the artificial *Tragopogon* with similar morphometry and descent speed. We use 3D printing of pure cellulose acetate (CA) in acetone for the fabrication of a thin porous pappus (≈20 µm thick), as the CA is biodegradable^[^ ^20^ ^]^ and the printing process is well established.^[^ ^21^ ^]^

CA is a linear polysaccharide that refers to any acetate ester of cellulose^[^ ^22^ ^]^ and we choose it as polymer for the fabrication of the artificial *Tragopogon* for its suitable mechanical properties, processability through additive manufacturing techniques, and biodegradation properties. CA is insoluble in water, but it is highly soluble in organic solvents (e.g., acetone until 30% w/v), and it has a Young modulus of 1.5 GPa.^[^ ^23^ ^]^ The first step, in the biodegradation, is the hydrolysis of the acetyl groups which requires the action of microorganisms with esterase, while the cellulose backbone is biodegraded by organisms with cellulase enzymes.^[^ ^20^ ^]^ Biodegradation times are highly variable depending on the degree of substitution (DS) of CA and on environmental conditions (humidity, temperature, and microorganisms).^[^ ^20^ ^]^ In laboratory scale‐compost conditions (53 °C), micrometric CA films disappeared in 30 days.^[^ ^24^ ^]^ Biodegradation tests with VGF (Vegetable, Garden, and Fruit) waste proved that pure cellulose acetate biodegraded completely within 200 days of testing after reinoculation of VGF.^[^ ^25^ ^]^ Similarly, CA fibers are reported to be biodegradable in moist soil, and they deteriorated after 2 months and destroyed after 4–9 months.^[^ ^20^ ^]^ Toxicity experiments in rats revealed that after a maximum oral administration of 5000 mg kg^−1^ body weight/day for 94–96 days no evidence of an adverse effect was recorded.^[^ ^26^ ^]^

In this study, CA was also used for the fabrication of a lightweight (≈20 mg) porous beak and for the matrix of colorimetric indicators (with micrometric pores) by coupling additive manufacturing and leaching techniques previously reported by our group^[^ ^27^ ^]^ to decrease the total weight of the integrated flier.

First, we proved the employment of the artificial *Tragopogon* for perspective aerial seeding by coupling the artificial beak with a mustard seed, as a proof of concept. Then, we also showed wireless monitoring by coupling the center of the pappus with colorimetric indicators for perspective measurement of rain pH^[^ ^28^ ^]^ and nitrides in soil.^[^ ^29^ ^]^

Our porous artificial *Tragopogon* can inspire the development of sustainable, porous, and lightweight carriers able to travel for long distances and with an improved pay‐loading capability aimed to environmental preservation, such as environmental monitoring and seeding.

## 2 Results and Discussion

### 2.1 Morphometric and Aerodynamic Characterizations of *Tragopogon Pratensis* Fruits

**Figure 1:**
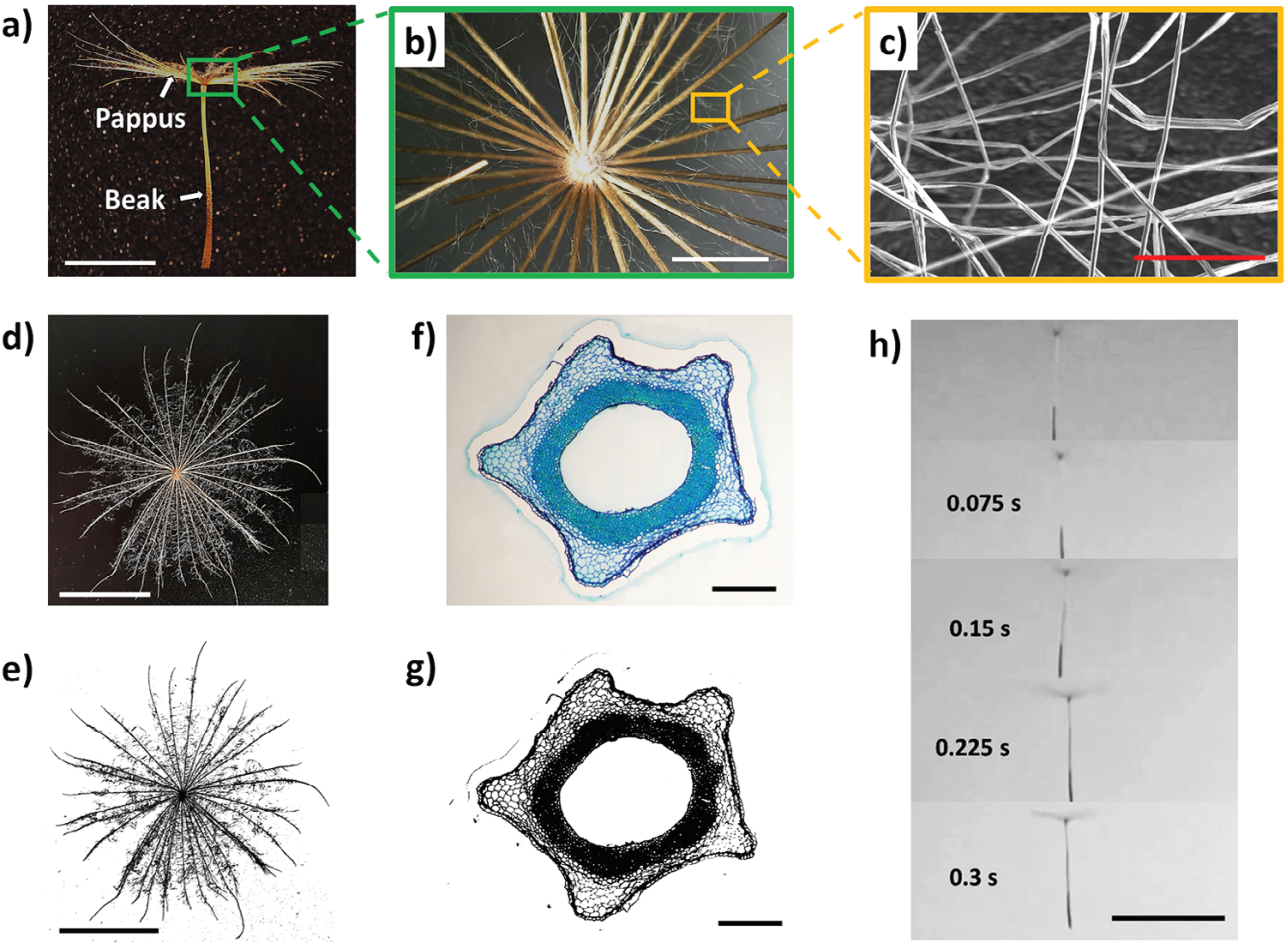
Morphometric and aerodynamic characterizations of *Tragopogon pratensis* fruit. a) Picture of a *Tragopogon pratensis* fruit showing beak and pappus. Scalebar is 2 cm. b) Optical microscope image of the central section of a pappus showing ribs. Scalebar is 2 mm. c) Dual beam image showing hairs of the pappus. Scalebar is 100 µm. d) Frontal view of the pappus showing ribs and hairs. Scalebar is 2 cm. e) Image binarization of the image reported in picture (d) for pappus projected porosity (*P*_p_, %) and surface (A, mm^2^) estimation. Scalebar is 2 cm. f) Optical microscope image of *Tragopogon pratensis* section of the beak at half of its length stained with Toluidine Blue. Scalebar is 200 µm. g) Binarization of the image reported in picture (f) for the beak porosity (*P*_b_, %) value calculation. The digital processing was performed with ImageJ.^[^ ^30^ ^]^ Scalebar is 200 µm. h) Overlapped frames of a free fall of a *Tragopogon pratensis* fruit taken from Video S1 (Supporting Information). Scalebar is 5 cm.

Natural *Tragopogon pratensis* fruits were collected and analyzed as a basis of our approach to bioinspired design and modeling of an artificial *Tragopogon*. The *Tragopogon pratensis* fruit (**Figure** **1**a) was composed of a pappus (i.e., the parachute) and a beak which embeds the embryo for germination (the seed).

All relevant morphometric characteristics of a natural *Tragopogon pratensis* fruit are summarized in Figures S1 and S2 (Supporting Information). The average mass was 28.4 ± 1.7 mg (*N* samples = 9), 16.5% ± 2.3% of which was in the pappus (*N* samples = 9). The pappus was composed of ribs and hairs (Figure 1b,c), that have the main role of maximizing the drag during the free fall.

The projected area of the whole pappus (A) is a key morphometric parameter for the estimation of the Drag Coefficient (*C*_D_)^[^ ^15^ ^]^ and it was estimated from pappus image capturing and binarization (Figure 1d,e). The result of the measurement was A = 3.25 ± 0.86 ×10^−4^ m^2^ (*N* samples = 9).

We also estimated the projected porosity of the pappus from Figure 1e by black and white pixels counting. The result of the measurement was *P*_p_ = 85.9% ± 4.0% (*N* samples = 9) in good agreement with the same analysis of hairs at microscale level reported in Figure S2 (Supporting Information) (*P*_h_ = 84.9% ± 2.0%) and with the porosity previously reported in literature (i.e., 90.5% ± 0.9%).^[^ ^18^ ^]^

The *Tragopogon pratensis* beak was analyzed separately. Transversal sections of the long, slender beaks were cut and then stained with the histochemical dye Toluidine Blue (Figure 1f; and Figure S3, Supporting Information). Interestingly, the *Tragopogon* beak section has a ring shape, characterized by an internal layer of densely packed lignified fibrous cells, which most probably reinforces the ring, and an external layer of parenchyma‐like cells (Figure 1f). An estimation of the beak porosity (*P*_b_) section at half of its length was carried out after image binarization and black and white pixels counting (Figure 1g). The result of the measurement was *P*_b_ = 55.0 ± 2.1% (*N* samples = 3).

An aerodynamic analysis was necessary for the measurement of the flying properties of the fruit and for the realization of an artificial flier with similar flying properties.

The key parameters of the flight performance of *Tragopogon pratensis* are: the terminal velocity (U), the Reynolds number (Re), and the previously mentioned drag coefficient (*C*_D_).^[^ ^15^ ^]^ We determined U by releasing the fruit from rest in a still air setting from a height of 2.0 m and allowing them to fall freely. The mean U measured was 0.61 ± 0.06 m s^−1^ (*N* samples = 9). This value was used to compute Re according to its definition

(1) Re=UDν

being D, the mean value of the diameter of the pappus and ν the kinematic viscosity of the air (1.5 × 10^−6^ m^2^ s^−1^ at 20 °C).

As a result, a mean Re = 2306 ± 303 value was estimated (*N* samples = 9). Then, the drag was calculated accordingly, considering the mean mass (m), the gravity acceleration (g), the density of the air at 20 °C (*ρ* = 1.204 kg m^−3^), the projected surface of the pappus (A) previously calculated

(2) $$C_{D}=\frac{\mathrm{mg}}{\frac{1}{2}\rho AU^{2}}$$

As results a mean *C*_D_ = 3.82 ± 0.81 (*N* samples = 9) was obtained.

### 2.2 Design and Additive Manufacturing of the Artificial *Tragopogon*

A simplified scheme (Figure S4, Supporting Information) of the additive manufacturing process of the artificial *Tragopogon* involves: i) picture of *Tragopogon pratensis* fruit, ii) morphometric analysis, iii) creation of a 3D CAD model, and iv) fabrication of the artificial *Tragopogon*. 3D Printing through Direct Ink Writing (DIW) of pure CA, (25% w/w in acetone) was used for the pappus fabrication, while molding of CA mixed with lignin was employed for the fabrication of the porous beak and the disk junction, accordingly to the leaching technique previously reported by our group.^[^ ^27^ ^]^

**Figure 2:**
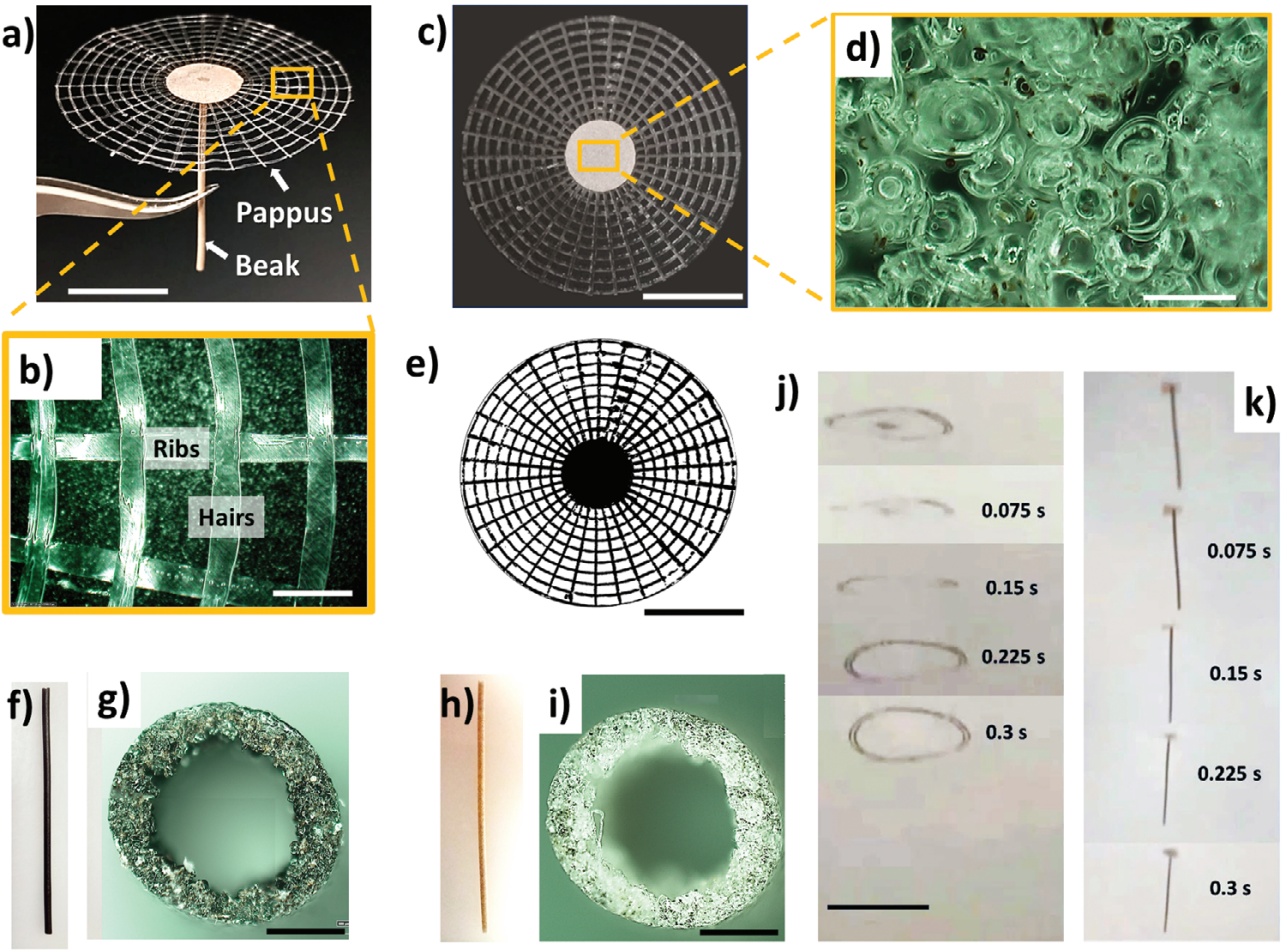
Design and additive manufacturing of the artificial *Tragopogon*. a) Picture of artificial *Tragopogon* showing beak and pappus. Scalebar is 2 cm. b) Optical microscope image of the 3D printed pappus in cellulose acetate showing ribs and hairs. Scalebar is 2 mm. c) Frontal image of the artificial pappus integrated with porous cellulose acetate disk junction. d) Optical microscope image of the porous disk junction surface after the leaching of lignin. Scalebar is 200 µm. e) Image binarization of the image reported in picture c) for pappus projected porosity (*P*_p_, %) and surface (A, mm^2^) estimation. Scalebar for (c) and (e) is 2 cm. f) Artificial beak made from the molding of a solution CA/Lignin/Acetone (20/20/60%w/w). g) Section of the cylindrical beak reported in (f) showing the hollow structure. h) The beak after the leaching of lignin. i) Section of the cylindrical leached beak reported in (h) showing the hollow structure. Scalebar for (g) and (i) is 500 µm. j,k) Overlapped frames of a free fall of an artificial pappus j) and artificial *Tragopogon* k) taken from Video S1 (Supporting Information). Scalebar is 5 cm.

The artificial *Tragopogon* structure, totally made in CA, is illustrated in **Figure** **2**a.

It consists of: a) a 3D printed radial concentric pappus, b) a porous beak in cellulose acetate realized through inject molding, c) a 3D printed porous disk junction for the connection between pappus and beak.

The 3D printed pappus was designed in agreement with the previously reported concentric design^[^ ^3^ ^]^ to emulate the structure of the natural pappus reported in Figure 1d. It was composed by 34 radial 3D printed artificial ribs with a length of 18.79 ± 0.04 mm and a width of 0.67 ± 0.06 mm (*N* samples = 24) and 9 circular circumferences with increasing radii from 9 to 20.5 mm and with a width of 0.62 ± 0.04 mm mimicking the hairs (*N* samples = 24) (Figure 2b,c). The average thickness of the ribs and hairs was 19.1 ± 2.7 µm (*N* samples = 6). The average mass of the pappus was 26.4 ± 2.2 mg (*N* samples = 10). All the morphometric features of the pappus are reported in Figure S5 (Supporting Information).

The disk junction was fabricated through 3D printing of a solution of CA/lignin/acetone (20/20/60% w/w). Alkali lignin leaching in water, as previously reported,^[^ ^27^ ^]^ provided lightening (mass reduction of roughly 50%) with the creation of a porous structure (Figure 2d). The porous junction, with mass 6.7 ± 0.5 mg (*N* samples = 14) and a porosity of 76.5 ± 1.1% (*N* samples = 14), was coupled to the artificial pappus using cellulose acetate in acetone as an adhesive (Figure 2c). The fabrication of pappus, porous beak, and disk junction are reported in Video S2 (Supporting Information). The used lignin alkali particles (83 ± 37 µm, *N* samples = 100, Figure S6, Supporting Information) generated micropores with an average size of 74 ± 33 µm (*N* samples = 100, Figure S7, Supporting Information).

The projected area of the whole artificial pappus (A) coupled with the porous junction was estimated from binarized pappus image (Figure 2e), as previously described. The result of the measurement was A = 12.13 ± 1.09 × 10^−4^ m^2^ (i.e., 1213 ± 109 mm^2^) (*N* samples = 6).

We also estimated the projected porosity of the pappus from Figure 2e by black and white pixels counting. The result of the measurement was *P*_p_ = 48.2 ± 4.7% (*N* samples = 9).

The porous beak was fabricated through molding of a solution of CA/lignin/acetone (20/20/60% in weight) using a cylindrical silicon tube as a mold, with an inner diameter of 1.3 mm and a length of 4.45 ± 0.04 (*N* samples = 6) mm (same length of the natural beak). Once the acetone evaporated and the silicone mold was removed, a black hollow cylinder was obtained due to the lignin black color (Figure 2f,g). The average mass of the hollow beak, like the natural one, was generated by the evaporation of acetone and the average mass was 43.7 ± 1.7 mg (*N* samples = 15) (Figure 2f,g). Then, the artificial beaks were leached, as described for the disk junction, and the mass was roughly halved to 20.7 ± 1.3 mg (*N* samples = 15), similarly to the natural beak one (24.2 ± 0.5 mg) (Figure 2h,i). Accordingly with calculations, the estimated porosity of the beak was 76.2 ± 0.4% (*N* samples = 6). The artificial beak was orthogonally coupled with the circular junction and pappus as previously described with cellulose acetate in acetone solutions as an adhesive.

The whole mass of the artificial *Tragopogon* was 53.8 ± 4.0 mg (*N* samples = 10). All the morphometric features of the artificial *Tragopogon* were reported in Figure S5 (Supporting Information).

From the aerodynamic point of view, the artificial pappus (without beak) and *Tragopogon* displayed an average descent speed U = 0.46 ± 0.03 m s^−1^ (*N* samples = 3) and 0.65 ± 0.05 m s^−1^ (*N* samples = 6), respectively. The descent speed of the artificial *Tragopogon* was statistically identical to the natural *Tragopogon pratensis* (i.e., 0.61± 0.06 m s^−1^, *N* samples = 9).

The calculated Re and C_D_ for the artificial pappus were 1599 ± 109 and 1.87 ± 0.32 (*N* samples = 3), respectively, while for the whole artificial *Tragopogon* were 2333 ± 202 and 1.55 ± 0.22 (*N* samples = 4), respectively.

In perspective, the estimated descent speed (U) could be used for the prediction of the traveling distance of an artificial pappus and *Tragopogon* over the wind using the following simple ballistic model already exploited in dispersal studies of natural and artificial seeds and fruit^[^ ^6^, ^31^, ^32^ ^]^

(3) $$x_{p}=H\frac{v_{w}}{U}$$

where *x*_p_ is the predicted traveling distance, *H* is the height of release, *v*_w_ is the wind velocity, *U* are the descent speeds of the artificial pappus and *Tragopogon* recorded indoor, assuming the absence of turbulences as the wind speed remained constant for the duration of the fall.

### 2.3 Mathematical Modeling

For an effective dispersal of the artificial *Tragopogon*, it is worth considering not only the terminal velocity during the free fall, but also the time needed to achieve it. Ideally, such value of velocity should be reached as fast as possible to maximize the descent time and hence the distance that the artificial *Tragopogon* can travel along the horizontal direction. To describe the dynamics of both the natural and the artificial *Tragopogon*, we have implemented a simple model. Denoting by *z*(*t*) the height at which the *Tragopogon* is located at time *t*, it is

(4) $$m\ddot{z}-\frac{1}{2}\rho AC_{D}{\dot{z}}^{2}=-mg$$

We first used the model to find *z*(*t*) and $\dot{z}\left(t\right)$ for the natural *Tragopogon* falling from a height *z*_0_ =  2 m. With the parameters reported in Figures S1 and S2 (Supporting Information), obtained experimentally, the model provides a terminal velocity ${\dot{z}}_{\mathrm{term}}=\;0.61$ m s^−1^ and a descent time equal to 3.32 s, as expected. The time needed to reach ${\dot{z}}_{\mathrm{term}}$ is smaller than 0.25 s.

For the artificial *Tragopogon*, as said in the previous section, the analysis of the experimental results provided us with *C*_D_ =  1.55. Accordingly, based on the model, the terminal velocity results equal to 0.68 m s^−1^ (reached in less than 0.21 s), and touches the ground in 2.97 s, in good agreement with the experimental evidence.

It must be noted that the drag coefficients of the natural and the artificial Tragopogon are significantly different. In view of future work aimed at artificial fliers with prescribed terminal velocity, we assessed whether the model could serve as a tool for design. For this scope, we extracted and fitted the data reported by^[^ ^3^ ^]^ in one of their Tables (Supporting Information) to obtain a polynomial expression *C*_D_(*Re*,*m*). In this way, we treated the drag coefficient as a function of the Reynolds number and of the total mass of the flier. The model with a variable *C*_D_ provides for the artificial *Tragopogon* a descent speed equal to 0.76 m s^−1^. Therefore, this model overestimates the velocity, underestimating the descent time: the result is ≈2.7 s.

From this implementation, we could draw two conclusions: i) both the artificial and the natural *Tragopogon* reach their terminal velocity in a relatively short time compared to the total descent time; and ii), the drag coefficient calculated experimentally for the *Tragopogon pratensis* cannot be used to model the dynamics of the artificial *Tragopogon*, and vice versa. In fact, we used the polynomial expression found for the variable *C*_D_ to model the dynamics of the *Tragopogon pratensis*, and the model turned out to be unsuitable: it provided a terminal velocity equal to 1.37 m s^−1^, notably greater than the value measured experimentally. In our opinion, this is due to the significant differences between the morphology of the two specimens: the natural *Tragopogon* fruit is provided with a relatively thick array of ribs surrounded by much thinner hairs, while the artificial pappus has concentric rings. Although we did not perform fluid dynamics simulations, we infer that the morphology of the pappus has a non‐negligible influence on the fluid dynamics around the *Tragopogon*, and that this is the reason for the great difference observed in their drag coefficients: 3.82 for the natural versus 1.55 for the artificial *Tragopogon*.

A consequence of ii) is that, unless further experimental characterizations are performed on artificial *Tragopogon* with different pappus morphologies, the function *C*_D_(*Re*,*m*) used in this work can be adopted only for the current geometry.

The solutions reported here have been obtained by numerical integration. All the details concerning the model, the implementation, and the results can be found in Figures S8 and S9 (Supporting Information).

### 2.4 Seeding and Colorimetric Monitoring with the Artificial *Tragopogon*

After assessing the aerodynamic performance in terms of descent speeds and modeling of the artificial *Tragopogon*, we coupled a mustard seed to the beak to demonstrate perspective applications in aerial seeding.

**Figure 3:**
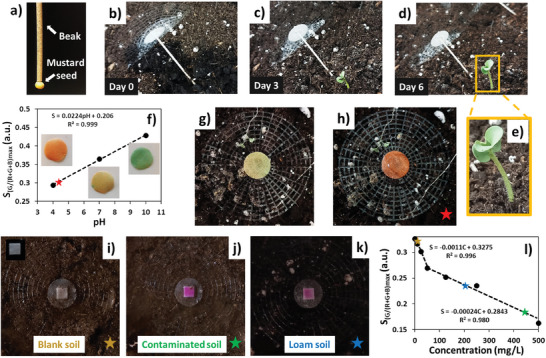
Seeding and colorimetric monitoring with the artificial *Tragopogon*. a) Artificial porous beak coupled with a mustard seed using Carboxymethyl Cellulose (CMC) as a biodegradable and water‐soluble adhesive. b) Self‐burial of the artificial *Tragopogon* coupled with a mustard seed promoted by water runoff. c) Germination of the mustard seed after 3 days of incubation. d) Growing of the mustard plant after 6 days of incubation. e) Zoom of the mustard plant taken from picture in (d). f) Colorimetric calibration of the 3D printed pH indicator using buffers (pH = 4, 7, and 10) accordingly with the RGB analysis reported in Figure S11 (Supporting Information). Red star shows the value of the pH after colorimetric analysis of the indicator integrated with artificial pappus reported in (g,h). h) pH indicator integrated with the artificial pappus on the soil. h) pH indicator color change after the dropping of a water acid solution (pH = 4.2). i–k) Commercial nitrate colorimetric indicator integrated with the artificial pappus on a i) noncontaminated soil (blank), nitrate‐contaminated soil j), and commercial loam soil k) after the addition of water (runoff simulation). Inset in (i) shows an indicator in deionized water. l) Colorimetric calibration of the nitrate indicator accordingly with the RGB analysis reported in Figure S11 (Supporting Information). Stars show the values of the nitrate concentration levels in soil samples i–k) interpolated after colorimetric analysis.

The mustard seeds were coupled to the end of the artificial beak using Carboxymethyl Cellulose (CMC) as a biodegradable and water‐soluble adhesive (**Figure** **3**a). A pool of five artificial *Tragopogon* coupled with mustard seeds (mass = 5.5 ± 1.0 mg, *N* samples = 10) were released from a height of 1 meter and dropped onto a model soil. Then, the burial was promoted using water mimicking rain or runoff (Figure 3b; and Video S3, Supporting Information). After 3 days of incubation in the growth chamber, 100% of the mustard seeds germinated (Video S3, Supporting Information), proving the perspective employment for aerial seeding using, for example, unmanned aerial vehicles (UAV).

After 10 days of tests in growth chamber, we eradicated the grown‐up mustard plants and measured and compared the mass with mustard plants grown as control reference one, i.e., not coupled with artificial *Tragopogon* (Figure S10, Supporting Information). The mass values were 45.6 ± 6.8 and 43.4 ± 5.3 mg (*N* samples = 5) for coupled and not coupled plants, respectively. This proved that coupling between artificial *Tragopogon* and mustard seed had no negative effects on germination and plant growth.

In perspective, the artificial *Tragopogon* could be integrated with other bioinspired machines designed for aerial seeding, which could promote soil drilling driven by humidity.^[^ ^8^, ^9^ ^]^ In addition, the artificial *Tragopogon* could be integrated with actuators, which modulate the angles between pappus and beak under the stimulus of humidity, as observed in the natural *Tragopogon pratensis* ^[^ ^18^ ^]^ and Dandelion,^[^ ^33^ ^]^ or under light stimulus as reported for the artificial *Dandelion* examples.^[^ ^34^, ^35^ ^]^ This would allow the modulation of the descent speeds according to the variation of environmental physical parameters.

Moreover, we investigated the artificial *Tragopogon* for environmental monitoring applications, since colorimetric sensors/indicators integrated with biodegradable *Tragopogon*‐inspired fliers can be attractive for “wireless” remote evaluation of environmental parameters via digital image capture and quantitative color extraction.^[^ ^4^ ^]^ For example, colorimetric sensing of pH could be significant for evaluating the acidity of rainfall,^[^ ^28^ ^]^ while monitoring nitrate levels could be valuable in assessing contaminated groundwater.^[^ ^29^ ^]^

For pH monitoring, a pH universal indicator was dissolved in a solution of cellulose acetate/NaHCO_3_/acetone, then 3D printed into the shape of a disk and made porous using the leaching technique previously described (Video S2, Supporting Information).^[^ ^27^ ^]^ In this case, we used NaHCO_3_ crystals as the porogen material to be leached. NaHCO_3_ generated larger pores 151 ± 47 µm (*N* samples = 100) than lignin (Figure S7, Supporting Information), since it is composed of larger crystals with size of 108 ± 53 µm (*N* samples = 100) (Figure S6, Supporting Information). After leaching, the indicator disk mass was 6.8 ± 0.07 (*N* samples = 5) mg with an estimated porosity of 55.3 ± 3.1% (*N* samples = 5).

The pH indicator was calibrated with buffers at pH (4–10) using a colorimetric analysis previously reported,^[^ ^36^ ^]^ plotting the normalized values of Green (G/(R+G+B)) (Figure 3f) and obtaining a good linearity (*R* ^2^ = 0.999).

Then, the indicator was coupled to the artificial pappus, using cellulose acetate solution as adhesive, and released on a model soil (Figure 3g). Subsequently, droplets of an aqueous acid solution at pH 4.2 were dropped onto the pH indicator, mimicking a case of acid rain. The color changed from yellow to red (Figure 3h) and, through the previously reported calibration, it was possible to trace a pH value of 4.1, thus proving an accuracy roughly of 98%. The approach for the pH indicator, although based on the integration of universal pH indicators in the CA porous structure with micrometric pores, is very flexible. In principle, any other molecule color‐sensitive to any physical and chemical parameter could be integrated within the porous structure,^[^ ^37^, ^38^ ^]^ through 3D printing.

As a proof of concept of nitrate monitoring, we coupled a commercial colorimetric indicator to the porous junction section using cellulose acetate solution as adhesive. The artificial pappus was released on different model soils (i.e., soil with poor nitrate levels (Figure 3i), soil with high level of nitrate contamination (1000 mg kg^−1^) (Figure 3j),^[^ ^39^ ^]^ and commercial loam soil (Figure 3k). Details of soil sampling, preparation and colorimetric analysis are reported in the Supporting Information section. After a simulation of runoff, different violet color intensities were recorded in agreement with the different amount of nitrate release. The different colors were analyzed with the colorimetric calibration fitting reported in Figure 3l. As a result, the releases of nitrate from soil to water were: 8.1 mg L^−1^ for blank soil; 446 mg L^−1^ for contaminated soil and 210 mg L^−1^ for loam soil. In the case of contaminated soil, we spiked 15 g of blank soil with 1000 mg kg^−1^ of sodium nitrate, and we leached with 30 mL of deionized water. According to the calculations, the nitrate release should have been 500 mg L^−1^. We got 446 mg L^−1^ by the performed colorimetric analysis, showing a satisfactory accuracy of about 90%.

While these measurements provide the concentration dissolved in water rather than the actual concentration of nitrates in the soil, we believe that they can be useful for a preliminary qualitative and wireless assessment of the soil's aridity, fertility, and contamination.

Once established the impact of the artificial *Tragopogon* both in the field of aerial seeding and environmental monitoring, we provide below some considerations concerning the environmental stability of the cellulose acetate to chemical degradation, accordingly with the current scientific literature. The stability of cellulose acetate (CA) when exposed to some atmospheric agents, such as water and sunlight, was previously reported.^[^ ^20^, ^40^ ^]^

Yadav et al. (2022) monitored the aging of CA in different aqueous environments, including lake water, seawater, and artificial seawater. Over 12 months, they observed a mild degradation of the material, with weight loss ranging from 5% to 10%, depending on the environment and temperature.^[^ ^40^ ^]^ Regarding light exposure, CA is photochemically degraded by UV wavelengths shorter than 280 nm but exhibits limited photodegradability in sunlight due to the absence of chromophores that absorb ultraviolet light.^[^ ^20^ ^]^ However, the addition of titanium dioxide, used as a whitening agent in various consumer products, such as the food coloring additive,^[^ ^41^ ^]^ could significantly enhance CA photodegradability.^[^ ^20^ ^]^ The addition of agents able to improve photodegradability could be a strategy for further reducing environmental impact in the case of large‐scale release of artificial *Tragopogon* fliers.

## 3 Conclusions

In summary, we have reported the design, modeling, and development of an artificial, biodegradable, porous, and parachute flying fruit for perspective environmental preservation, such as wireless colorimetric monitoring of environmental parameters and aerial seeding. The artificial *Tragopogon* resembled the morphometry and aerodynamic features of the natural *Tragopogon pratensis* parachute fruit.

We produced the artificial *Tragopogon* by coupling 3D printing, molding, and leaching of cellulose acetate (CA): a biodegradable and bioderived polymer.^[^ ^20^ ^]^

The artificial pappus was coupled with a 3D‐printed porous and colorimetric pH indicator in CA, realized through leaching technique, and with a commercial nitrate indicator for perspective monitoring of pH rain or nitrate level in soil after runoff, respectively. Compared to the current state of the art of seed‐inspired fliers integrated with sensors/indicators, this work reports a flier integrated with colorimetric sensors/indicators, made with biodegradable materials and inspired by the parachuting fruit of *Tragopogon pratensis*. The bioinspiration and design choice enables the development of a flier with one of the lowest falling speeds (0.46 m s^−1^) coupled with one of the largest surface areas for the sensors/indicators (≈150 mm^2^) (Figure S12, Supporting Information). This could allow at the same time greater dispersion by the wind and easier reading, in perspective performed by a drone's camera. Due to the unique morphology of *Tragopogon pratensis* and the presence of a beak that encloses the seed, the bioinspired artificial flier can also be adapted to carry seeds from other species for aerial seeding, making it suitable for multipurpose applications.

We reported application in aerial seeding using a mustard seed integrated with the artificial *Tragopogon*. Mimicking a falling and a runoff, 100% of the seeds germinated after 3 days of incubation.

Thanks to the bioinspired structure we have reported the first example of artificial flier for both environmental monitoring and aerial seeding purposes.

In the not‐too‐distant future, these artificial fruit or seed‐like fliers, integrated with multiple colorimetric indicators/sensors and/or natural seeds, could be released by drones equipped with camera technologies for monitoring environmental parameters or the growth of plants after aerial seeding.

## Conflict of Interest

The authors declare no conflict of interest.

## Supporting information



Supporting Information

Supplemental Video 1

Supplemental Video 2

Supplemental Video 3

## Data Availability

The data that support the findings of this study are available from the corresponding author upon reasonable request.
