# Supplementary material for: A Biodegradable, Porous Flier Inspired by a Parachute‐Like Tragopogon Fruit for Environmental Preservation
Source: Small. 2024 Sep 17;21(3):2403582. doi: 10.1002/smll.202403582 (PMC11753481; doi:10.1002/smll.202403582)
Supplement: Supplementary file 1 — Supporting Information [file SMLL-21-2403582-s001.docx]

Supporting Information

**A biodegradable, porous flier inspired by a parachute-like Tragopogon fruit for environmental preservation**

Stefano Mariani,^1^*^#^ Kliton Cikalleshi,^1,2,#^ Marilena Ronzan,^1^ Carlo Filippeschi, ^1^ Giovanna Adele Naselli^1^ and Barbara Mazzolai ^1^*

^1^Bioinspired Soft Robotics Laboratory, Istituto Italiano di Tecnologia, Via Morego 30, Genova 16163, Italy

^2^The Biorobotics Institute, Scuola Superiore Sant'Anna, 56025 Pontedera, Italy
E-mail: stefano.mariani@iit.it; barbara.mazzolai@iit.it

#These authors contributed equally

1. Experimental section

1.1 Morphometric and histological characterizations of the natural *Tragopogon pratensis* fruit

1.2 Determination of the aerodynamic performance of the natural *Tragopogon pratensis* fruit

1.3 3D printing process of the artificial *Tragopogon*

1.4 Morphometric and aerodynamic characterizations of the artificial *Tragopogon*

1.5 Modeling

1.6 Aerial seeding and germination experiments

1.7 Colorimetric monitoring experiments

1.8 Statistical analysis

Figure S1. Morphometric analysis of *Tragopogon pratensis* fruit

Figure S2. Morphometric analysis of the pappus

Figure S3. Lignin localization observed through autofluorescence in UV light with DAPI filter

Figure S4. Flow chart for the 3D printing/molding of the artificial *Tragopogon*

Figure S5. Morphometry of the artificial *Tragopogon*

Figure S6. Analysis of the porogen particles

Figure S7. Analysis of the pores

Figure S8. Modeling

Figure S9. Modeling

Figure S10. Weight comparison of coupled and not coupled mustard seeds.

Figure S11. Colorimetric analysis of pH and nitrate

Figure S12. State-of-the-art of seed-inspired fliers integrated with sensors for environmental monitoring

Video S1. Free fall of *Tragopogon pratensis*, artificial pappus, and artificial *Tragopogon*

Video S2. Fabrication of the artificial *Tragopogon*

Video S3. Aerial seeding, germination, and growing

**1. Experimental section**

**1.1 Morphometric and histological characterizations of the natural *Tragopogon pratensis* fruit**

*Tragopogon pratensis* fruits were collected in the rural area of Pescia (Pistoia, Tuscany, Italy). Morphometric analysis of the fruits was carried out using a digital caliper (RS PRO 150 mm Digital Caliper 0.0005 in, 0.01 mm, Metric & Imperial, United Kingdom) with a resolution of ±0.01 mm, a digital microscope (KH-8700, Hirox, Japan), and Helios NanoLab 600i Dual Beam Focused ion beam/field-emission SEM instrument (FEI, USA). The mass was measured with an analytical balance (KERN ABS-N, Germany) with a resolution of ±0.0001 g. The experimental data were collected from a pool of 9 fruits.

The length of the beak (L_b_), the thickness of the beak in the end and in proximity of the pappus (T_b1_ and T_b2_), and the length of the ribs in the pappus (L_r_) were measured with the caliper.

The thickness of the ribs (T_r_) was measured with the digital microscope while the thickness of the hairs (T_h_) was measured with the Dual Beam microscope.

Figure S1d summarize the values of the morphometric parameters.

The pappus projected surface (A) was estimated from pictures of *Tragopogon pratensis* pappi (9 fruits) captured with a camera (1280 × 800 pixels) of a Samsung A40 (South Korea) smartphone. The images were binarized using ImageJ^[1]^ and A was evaluated by counting the black pixels within a scale bar of 2 cm.

The pappus porosity projected (P_p_) was estimated from the binarized images of the pappi and by black and white pixels counting, representing the voids and the material, respectively and accordingly to the follow equation:

$$P_{p}=\frac{N_{white pix}}{N_{white pix}+N_{black pix}}$$

For the histological analysis *Tragopogon pratensis* beaks were embedded in resin by using Technovit ®7100 and sectioned at 8 µm thickness with a manual microtome (Leica SM2010R, Germany). The sections were stained with Toluidine blue (Merck, Germany) at 0.05% for 1.30 min, rinsed with deionized water.

The beak porosity (P_b_) in the thicker section was estimated from the optical microscope image. The image was processed with ImageJ^[1]^ and binarized. The porosity was estimated by black and white pixels counting, representing the voids and the material, respectively and accordingly to the previously reported equation.

**1.2 Determination of the aerodynamic performance of the natural *Tragopogon pratensis* fruit**

The analysis of aerodynamics of natural fruits under laboratory conditions consisted of the measurements of key parameters that characterize the flight, such as (i) descent speed (U). To determine the descent speed (U), the *Tragopogon pratensis* fruits were released from rest in a still air setting from a height of 2.00 m and allowed to fall freely. Tests were conducted in a laboratory without active ventilation. The flight of the fruits was recorded by a camera of a Samsung A40 (South Korea) smartphone. The mean U was calculated considering the time elapsed between the frame of the release and the frame in which the *Tragopogon pratensis* touches the ground.

**1.3 3D printing process of the artificial *Tragopogon***

*Materials and chemicals*

The chemical reagents used included: acetone 99.5%, cellulose acetate (30000 MW), sodium bicarbonate and carboxymethylcellulose sodium salt (CMC, high viscosity) purchased from Merck (Germany), lignin (Alkaline) purchased from TCI Europe N.V., (Japan), and Universal Indicator, pH Range 4-10, for pH Measurement purchased from Fisher Chemical (United States). Deionized water (DIW) was purified by Arium® advance EDI (Sartorius, Germany).

*3D printing of the artificial pappus*

A cellulose acetate batch solution was prepared by mixing in a beaker cellulose acetate in acetone at 25% w/w. The beaker was closed with Parafilm and aluminum foil and was put on a magnetic stirrer (Thermo Fisher Scientific Inc., USA) at 50 ℃ at 30 rpm for 1 h. The solution was used for 3D printing of the artificial pappus through Direct Ink Writing (DIW) with a 3D-Bioplotter (EnvisionTEC, USA and Germany). Printing temperature and build plate temperature were set at 20 ℃, printing speed was set at 80 mm/s, pressure 0.1 bar and the diameter of the used nozzle was 0.4 mm.

*3D printing of the disk junction*

The material for the 3D printing of the disk junction was prepared by mixing in a beaker cellulose acetate/lignin/acetone (20/20/60% w/w). Lignin was added to the solution as porogen accordingly with the leaching technique previously reported by^[2]^. The beaker was closed with Parafilm and aluminum foil and was put on a magnetic stirrer (Thermo Fisher Scientific Inc., USA) at 50 ℃ at 30 rpm for 1 h. The solution was used for 3D printing through the Direct Ink Writing (DIW) with 3D-Bioplotter (EnvisionTEC, USA and Germany). Printing temperature and build plate temperature were set at 20 ℃, printing speed was set at 40 mm/s, pressure 0.5 bar and the diameter of the used nozzle was 0.4 mm. Disk junctions (radius ~ 7 mm) were 3D printed then dried in oven (Vacutherm, Thermo Electron LED GmbH, Germany) at 70 ℃ for 30 min, to let all the acetone evaporate. Then the junctions were put in a plastic petri dish filled with deionized water and left for 3 times (30 minutes each) to allow the release of lignin in water. After each interval, the specimens were dried, first with adsorbent paper, then in oven (Vacutherm, Thermo Electron LED GmbH, Germany) at 70 ℃ for 30 min. Lignin particles and micropores sizes of the junctions were acquired with a digital microscope (KH-8700, Hirox, Japan) and analyzed using ImageJ.^[1]^

*Injection molding of the porous beak*

The material for the 3D printing of the porous beak was prepared by mixing in a beaker cellulose acetate/lignin/acetone (20/20/60% w/w). Lignin was added to the solution as porogen accordingly with the leaching technique previously reported by^[2]^. The beaker was closed with Parafilm and aluminum foil and was put on a magnetic stirrer (Thermo Fisher Scientific Inc., USA) at 50 ℃ at 30 rpm for 1 h. The solution was used for injection molding with a 3D-Bioplotter (EnvisionTEC, USA and Germany) in a silicon tube as mold with an inner diameter of 1.3 mm and a length of 4.5 mm. Pressure was 2.0 bar and the diameter of the used nozzle was 0.4 mm. The samples were dried in oven (Vacutherm, Thermo Electron LED GmbH, Germany) at 70 ℃ for 30 min, to let all the acetone evaporate. Then the silicone mold was removed with a lancet, and the beak was put in a plastic petri dish filled with deionized water and left for 3 times (30 minutes each) to allow the release of lignin in water. After each interval, the specimens were dried, first with adsorbent paper, then in oven (Vacutherm, Thermo Electron LED GmbH, Germany) at 70 ℃ for 30 min.

*3D printing of the pH porous sensor*

The material for the 3D printing of the porous pH sensor was prepared by mixing in a beaker cellulose acetate/NaHCO_3_/pH indicator/acetone (20/20/15/45% w/w). NaHCO_3_ was added to the solution as white porogen accordingly with the leaching technique previously reported by ^[2]^. The beaker was closed with Parafilm and aluminum foil and was put on a magnetic stirrer (Thermo Fisher Scientific Inc., USA) at 50 ℃ at 30 rpm for 1 h. The solution was used for 3D printing through the Direct Ink Writing (DIW) process with 3D-Bioplotter (EnvisionTEC, USA and Germany). Printing temperature and build plate temperature were set at 20 ℃, printing speed was set at 40 mm/s, pressure 0.5 bar and the diameter of the used nozzle was 0.4 mm. Disk samples (radius ~ 7 mm) were 3D printed. Then the junctions were put in a plastic petri dish filled with deionized water and left for 3 times (30 minutes each) to allow the release of NaHCO_3_ in water. After each interval, the specimens were dried, first with adsorbent paper, then in oven (Vacutherm, Thermo Electron LED GmbH, Germany) at 70 ℃ for 30 min. NaHCO_3_ particles and micropores sizes of the colorimetric indicators were acquired with a digital microscope (KH-8700, Hirox, Japan) and analyzed using ImageJ.^[1]^

*Coupling of the artificial Tragopogon*

The following coupling were realized using cellulose acetate in acetone as biodegradable adhesive (25% w/w):

- Artificial pappus, artificial porous beak and disk porous junction for the artificial *Tragopogon* fabrication.
- Artificial pappus and porous pH sensor
- Artificial pappus, porous disk junction and commercial colorimetric sensors

For the aerials seeding purposes mustard seeds (Franchi Sementi, Italy) were coupled with the artificial beak using carboxymethylcellulose sodium salt (CMC, high viscosity, Merck, Germany) (3% w/w in deionized water) as water-soluble and biodegradable adhesive.

**1.4 Morphometric and aerodynamic characterizations of the artificial *Tragopogon***

Morphometric and aerodynamic characterizations of the artificial pappus and *Tragopogon* were conducted within the laboratories as described in the previous Section 1.1 for the natural *Tragopogon pratensis*.

**1.5 Modeling**

The implementation has been performed in MATLAB® R2023b. The differential equation was solved numerically by means of the solver *ode45*. Results for the cases with constant drag coefficient are shown in Figure S8.

Values of the drag coefficient extracted from^[3]^ are fitted by a polynomial expression as follows:

$$C_{D}(Re,m)=p_{00}+p_{10}\frac{Re}{\tilde{Re}}+p_{01}\frac{m}{\tilde{m}}+p_{20}\left( \frac{Re}{\tilde{Re}} \right)^{2}+p_{11}\frac{Re}{\tilde{Re}}\cdot\frac{m}{\tilde{m}}+p_{02}\left( \frac{m}{\tilde{m}} \right)^{2}$$

with $p_{00}=2.1378$, $p_{10}= -1.6513$, $p_{01}=0.9680$, $p_{20}=0.4430$, $p_{11}=-0.3698$, $p_{02}=-0.0328$, $\tilde{Re}=2105$, $\tilde{m}=5.155\cdot{10}^{-5}$.

The fitting has been performed through the fitting tool within Matlab. The polynomial expression has been used to obtain the results shown in Figure S9.

For all the natural and artificial *Tragopogon*, we set as initial conditions $z_{0}=2$ m and $\dot{z}_{0}=0$ m/s.

**1.6 Aerial seeding and germination experiments**

Five mustard seeds (Il Sovescio, Franchi Sementi, Italy) were coupled to the end of the artificial beak of five artificial Tragopogon using one drop of Carboxymethyl cellulose (CMC, 3% w/w in deionized water) as a biodegradable adhesive. The coupled seeds were dried at 20 °C for 1 hour in room air. Then the five coupled artificial *Tragopogon* were released from a height of 1 meter and dropped onto a model loam soil (Terriccio Universale, Coop, Italy). Once they felt to the soil the burial was promoted using 20 mL of tap water mimicking rain or runoff. The germination experiments were performed in the growth chamber (temperature 25°, 60% humidity) and recorded using time-lapse photography at 5 minutes interval with a camera (Logitech, Swiss). The monitoring of germination/growing experiments was 6 days. Control experiments were carried out using five seeds burial in the same manner and incubated in the same conditions. After 10 days of growing, the mass of the mustard plants (coupled with the artificial Tragopogon and not) was measured with an analytical balance (KERN ABS-N, Germany).

**1.7 Colorimetric monitoring experiments**

The colorimetric and porous pH sensor was 3D printed as previously reported in *3D printing of the pH porous sensor* section. The sensor was calibrated using commercial buffers (pH= 4, 7 and 10). Color images of the sensor surface were captured with a smartphone camera (Galaxy A13, Samsung, South Korea). RGB values were extracted using ImageJ^[1]^ and normalized as reported in^[4]^. The normalized R/(R+G+B) values were plotted against the pH values. Then the sensor was coupled to the artificial pappus using CA solution in acetone (25% w/w) as biodegradable adhesive. The coupled artificial pappus was released and dropped onto a model commercial loam soil. Once it felt to the soil, 10 mL of apple vinegar (pH = 4.2) were dropped onto the pH sensor surface for mimicking acid rain. The color was analyzed after 30 minutes, as previously reported, and the corresponding pH value was interpolated from the calibration fitting. Colorimetric nitrate sensors (Drinking Water Tester, Ausyde) were coupled to the junction of the pappus using CA solution in acetone (25% w/w) as biodegradable adhesive. The mass was 3.8 ± 0.4 mg, (N samples = 6). Color images were acquired and elaborated with ImageJ as previously reported. The coupled artificial pappus was released and dropped onto 15 g of poor-nitrate soil sampled in an uncultivated area away from trees, contaminated soil (1000 mg/kg) after the spike of sodium nitrate solution (Merck, Germany), and commercial loam soil. Once it felt to the soil 30 mL of deionized water were added to the soil for the phase transfer from soil to the sensor surface. The test lasted 30 minutes. The color was analyzed as previously reported in^[4]^ and the corresponding nitrate concentration level was interpolated from the calibration fittings.

**1.8 Statistical analysis**

Where specified, data are provided as average values over N replicates with error bars representing one standard deviation. The N samples is indicated in the parentheses.


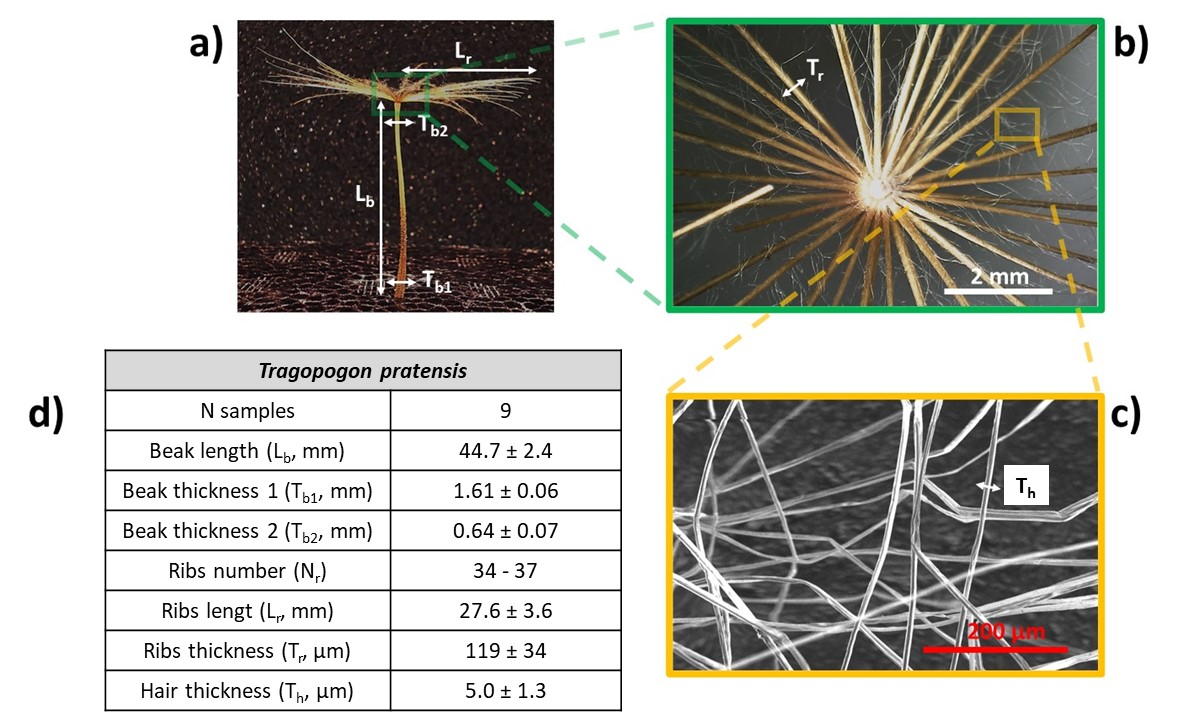


**Figure S1**. **Morphometric analysis of Tragopogon pratensis fruit.** Picture of a Tragopogon pratensis fruits with morphometric parameters where: L_b_ is the length of the beak; T_b1_ and T_b2_ are the thickness of the beak in the end and in proximity of the pappus; L_r_ is the length of the ribs in the pappus. b) Optical microscope image of the pappus of the Tragopogon pratensis where T_r_ is the thickness of the ribs. Scalebar is 2 mm. c) Dual Deam microscope image of the hair ribs where T_h_ is the thickness. Scalebar is 200 µm. d) Table summarizing the values of the morphometric parameters reported in (a-c).


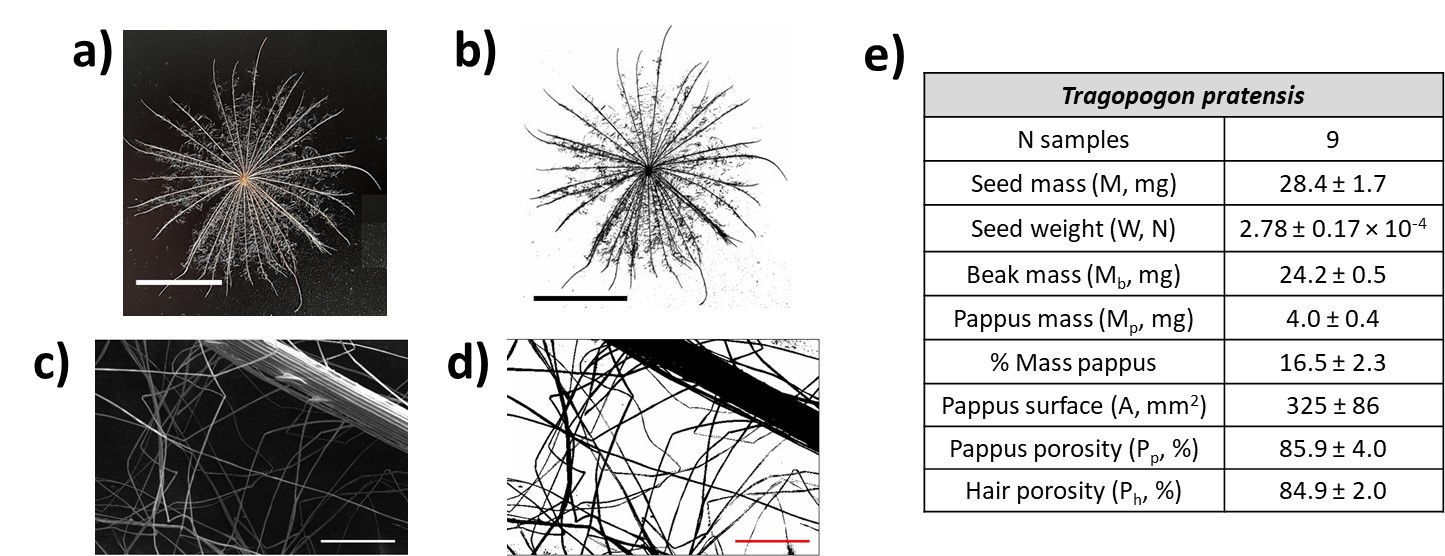


**Figure S2**. **Morphometric analysis of the pappus.** Picture of a Tragopogon pratensis pappus with ribs and hairs. b) Image binarization of the image reported in picture (a) for the surface and projected porosity evaluation. Scalebar is 2 cm. c) Dual Beam image showing a rib and hairs. Scalebar is 200 µm. d) Image binarization of the image reported in picture c) for the surface and projected porosity evaluation at microscopic scale. Scalebar is 200 µm. e) Table summarizing mass and weight values, pappus surface and porosity.


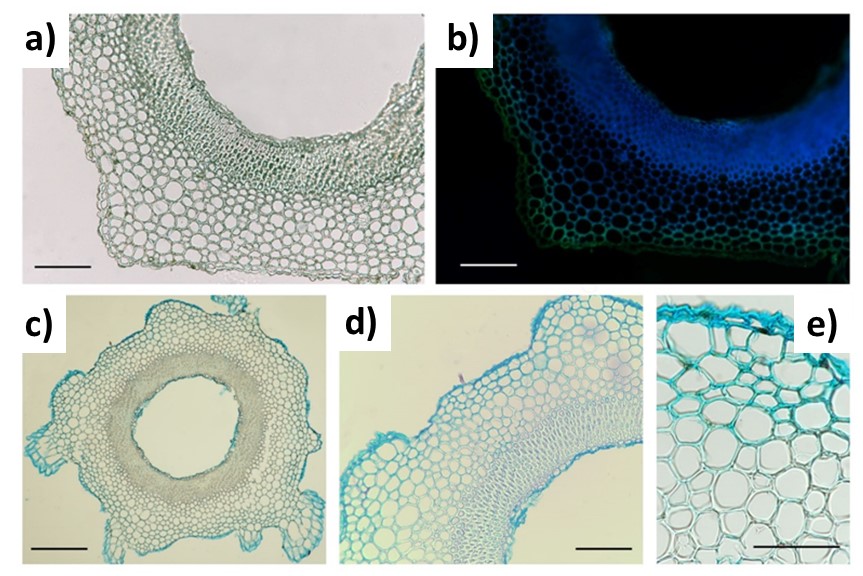


**Figure S3. Lignin localization observed through autofluorescence in UV light with DAPI filter**. a) Whole section of the beak in white light and b) in auto-florescence. c) White image of a detail of the fibrous ring region and d), it’s corresponding auto-florescence image. Scale is 100 µm in (a) and (b), and 50 µm in (c) and (d).

**
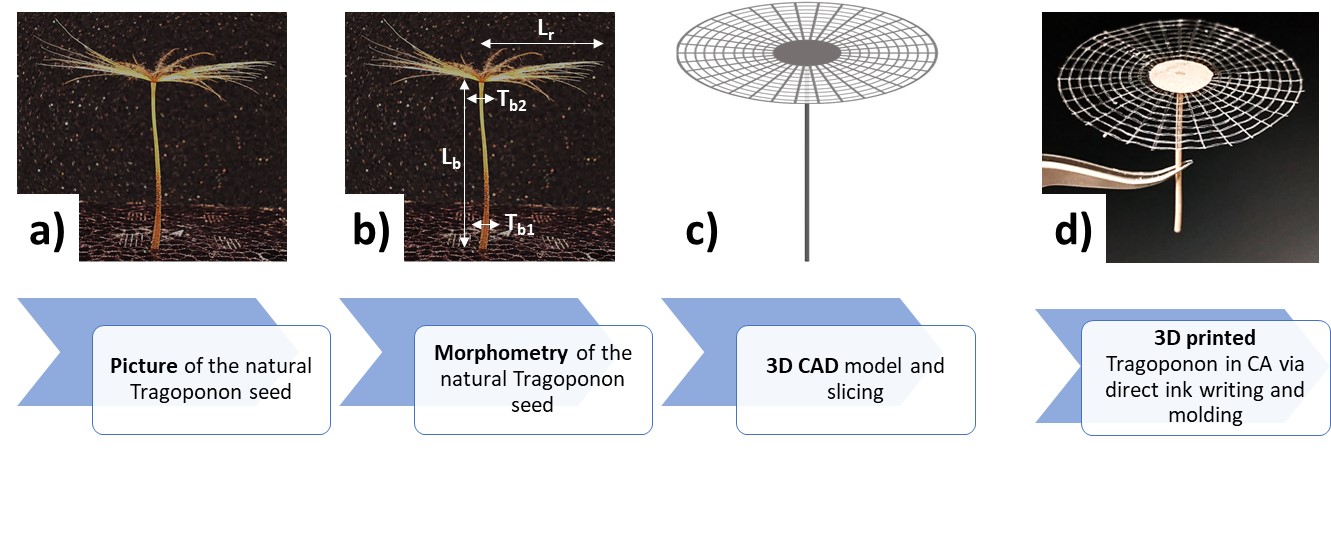
**

**Figure S4. Flow chart for the 3D printing/molding of the artificial Tragopogon pratensis** **fruit**. It consists of: a) picture of a Tragopogon pratensis fruit; b) morphometric analysis of Tragopogon pratensis fruit; c) creation of a 3D CAD model and slicing; d) 3D printing of the artificial pappus with CA and coupling of the porous artificial beak fabricated through molding of CA blended with lignin and leaching technique.


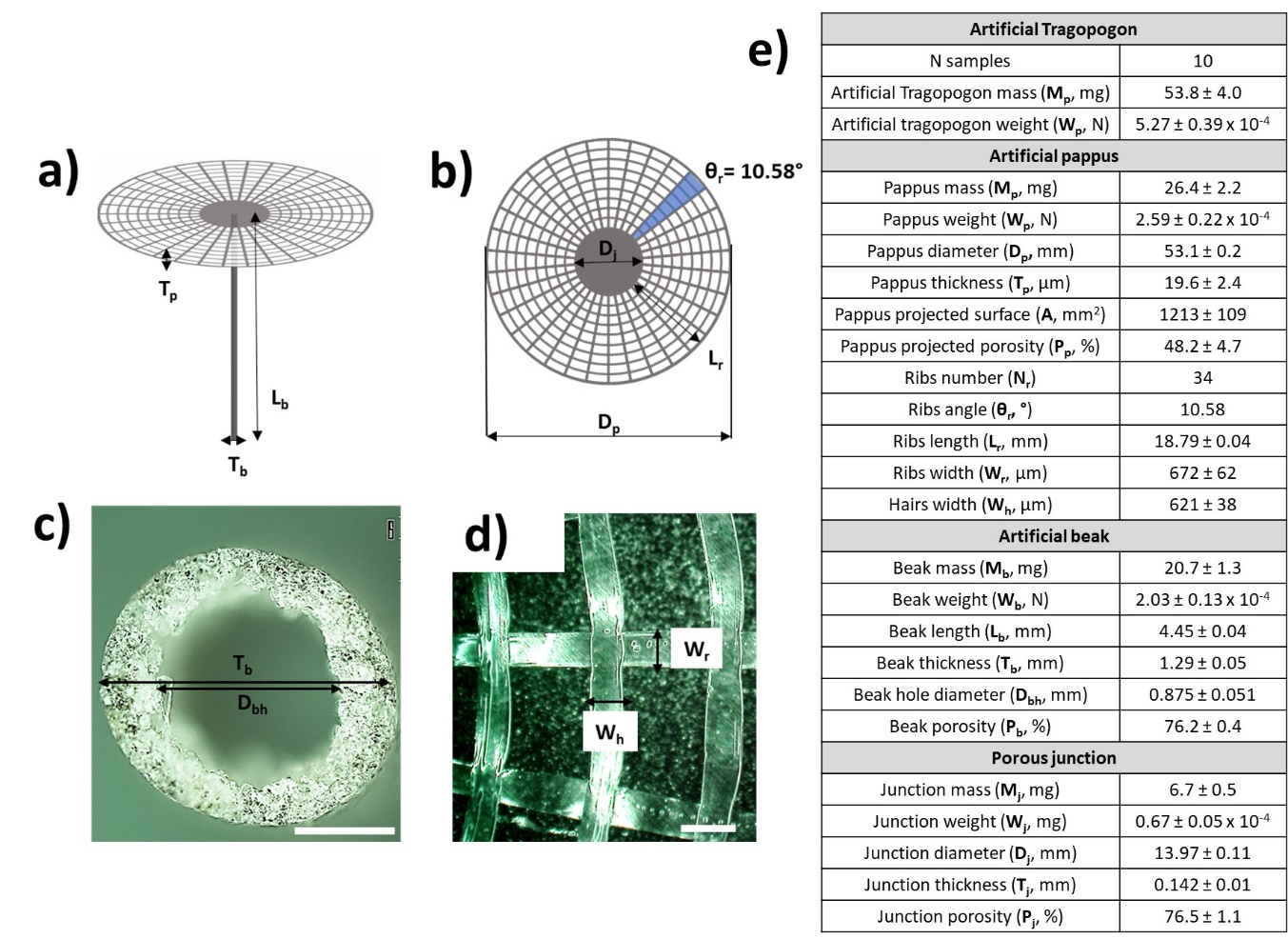


**Figure S5. Morphometry of the artificial *Tragopogon*.** a) Sketch of an artificial *Tragopogon* with morphometric parameters where: L_b_ is the length of the beak; T_b_ is the thickness of the beak; T_p_ is the thickness of the pappus. b) Sketch of a pappus of the artificial *Tragopogon* where: D_p_ is the diameter of the pappus; L_r_ is the length of the ribs; D_j_ is the diameter of the porous junction; θ_r_ in the angle between ribs. c) Picture of the disk porous section of the artificial beak showing the hollow structure where: T_b_ is the thickness of the beak; D_bh_ is the diameter of the hole. d) Zoomed picture of the artificial pappus showing ribs and hairs where: W_r_ is the width of the artificial ribs; W_h_ is the width of the artificial hairs. Scalebar is 1 mm. e) Table summarizing the values of the morphometric parameters of the artificial Tragopogon.


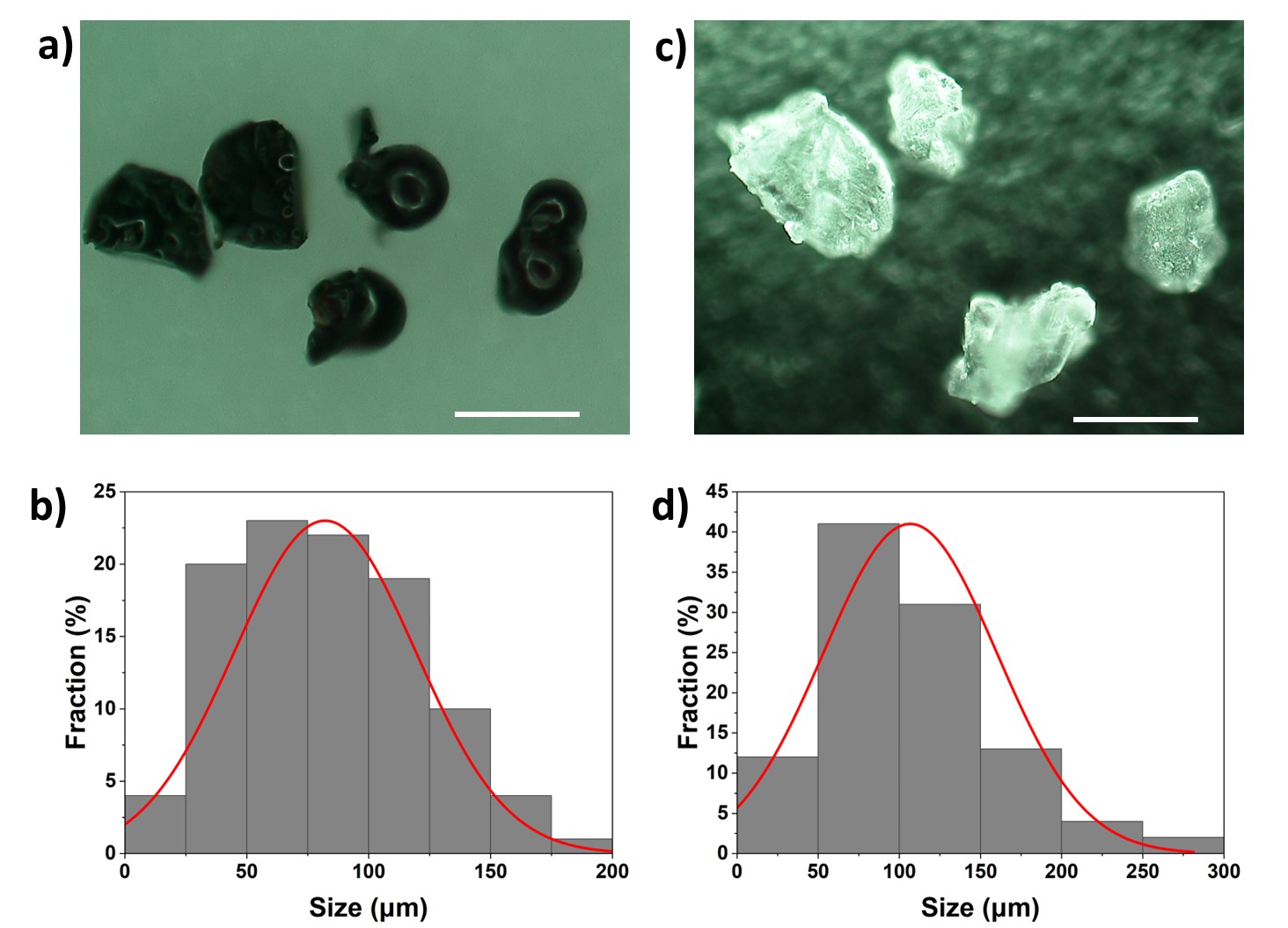


**Figure S6. Analysis of the porogen particles.** a) Particles of lignin alkali. Scalebar is 100 µm. b) Size distribution of the lignin particles (N samples = 100). c) Crystals of NaHCO_3_. Scalebar is 100 µm. d) Size distribution of NaHCO_3_ crystals (N samples = 100). Distribution analysis was performed with Origin (data analysis software) (OriginLab Corporation).

**
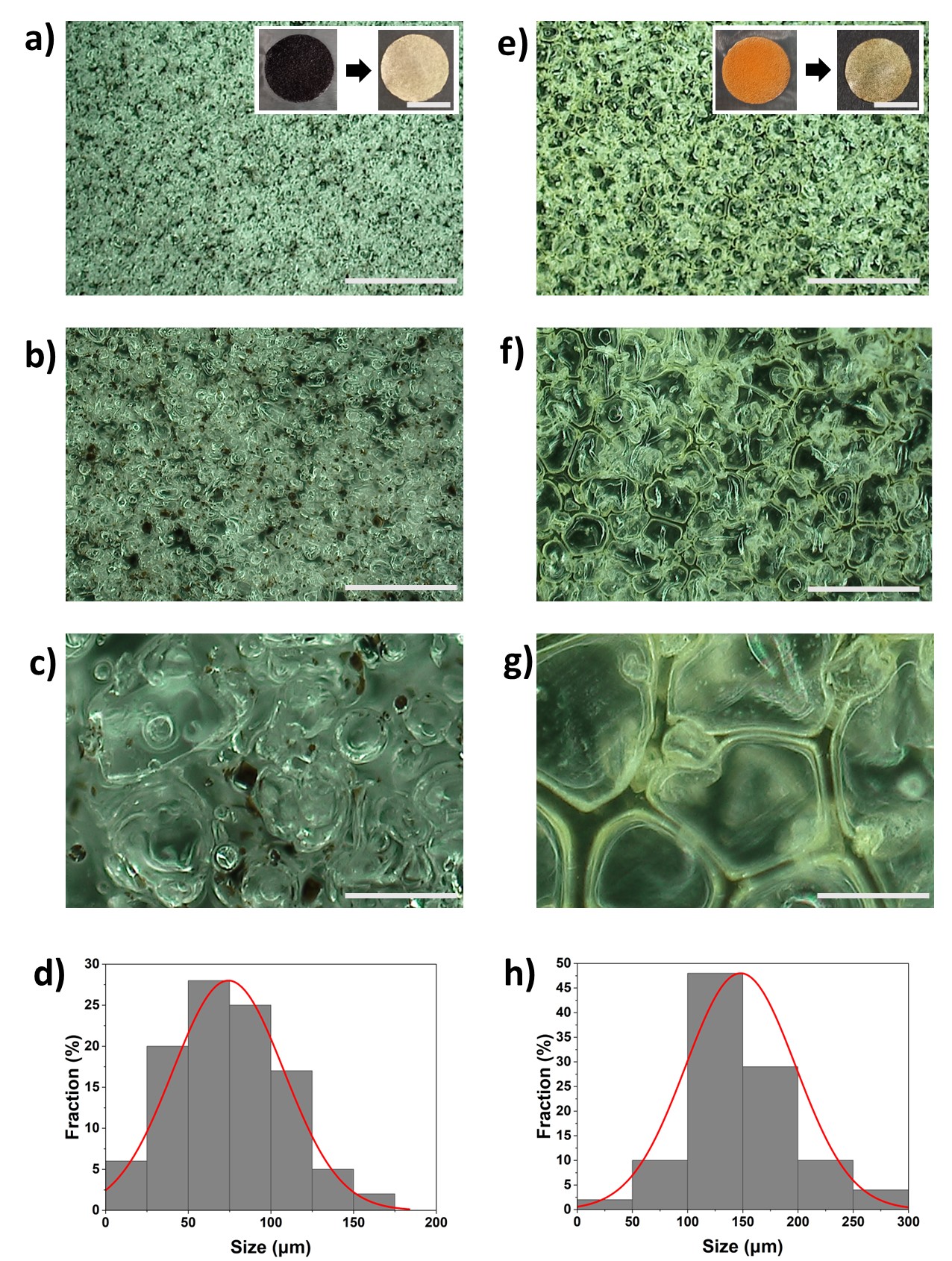
**

**Figure S7. Analysis of the pores.** a-c) Optical microscope image of the junction surface after leaching of lignin. Scalebar are 1 mm, 500 µm and 100 µm, respectively. Inset in (a) show a picture of the porous junction before and after the leaching. Scalebar is 1 cm. d) Size distribution of the micropores of the porous junction generated after the leaching of lignin particles. e-g) Optical microscope image of the pH sensor surface after leaching of NaHCO_3_ crystals. Scalebar are 1 mm, 500 µm and 100 µm, respectively. Inset in (e) shows a picture of the porous pH sensor before and after the leaching. Scalebar is 1 cm. h) Size distribution of the micropores of the porous junction generated after the leaching of NaHCO_3_ crystals. Distribution analysis was performed with Origin (data analysis software) (OriginLab Corporation).


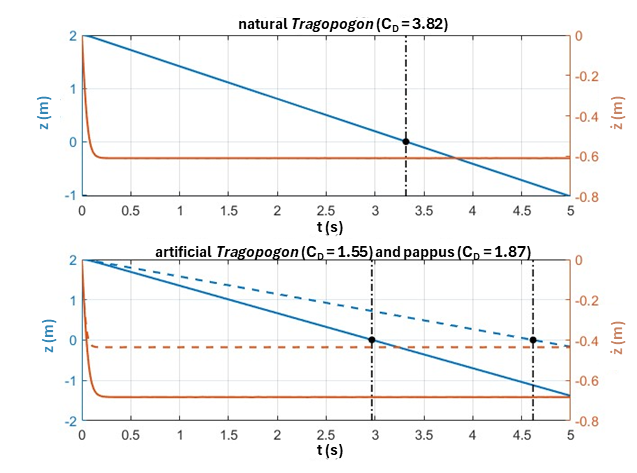


**Figure S8. Modeling.** Numerical solution obtained for the *Tragopogon pratensis* fruit (top) and the artificial *Tragopogon* (bottom) with the drag coefficient determined experimentally. For the natural *Tragopogon*, mass and area are as reported in Figure S2e. For the artificial *Tragopogon*, solid lines refer to the entire *Tragopogon*, dashed lines only to its pappus. In both plots, blue lines represent the descent vs. time, red lines represent velocities vs. time. The black dots and the black dash dotted lines mark the time at which the *Tragopogon* reaches the ground ($z=0$ m). The mass and the area are given the values reported in Figure S5e.

**
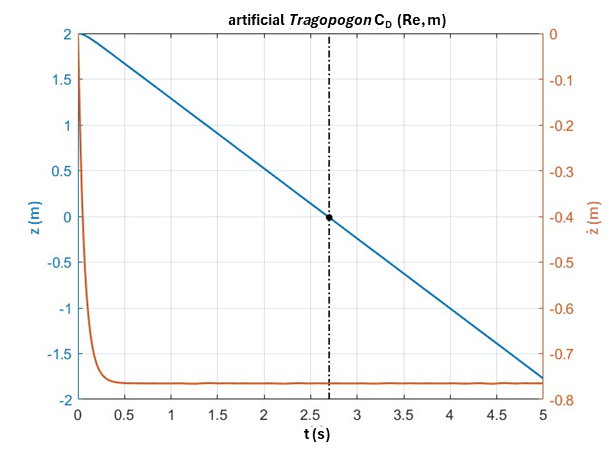
**

**Figure S9. Modeling.** Numerical solution obtained for the artificial *Tragopogon* with drag coefficient given by $C_{D}\left( Re,m \right).$ Blue and red lines represent descent vs. time and velocity vs. time, respectively.

**Figure S10.** **Weight comparison of coupled and not coupled mustard seeds**. a) Picture of germinated mustard plants (coupled and not coupled with artificial Tragopogon) after 10 days of incubation in growth chamber. b) Mass of mustard plants (coupled and not coupled with artificial *Tragopogon*) after 10 days of incubation in growth chamber (N samples = 5).

**
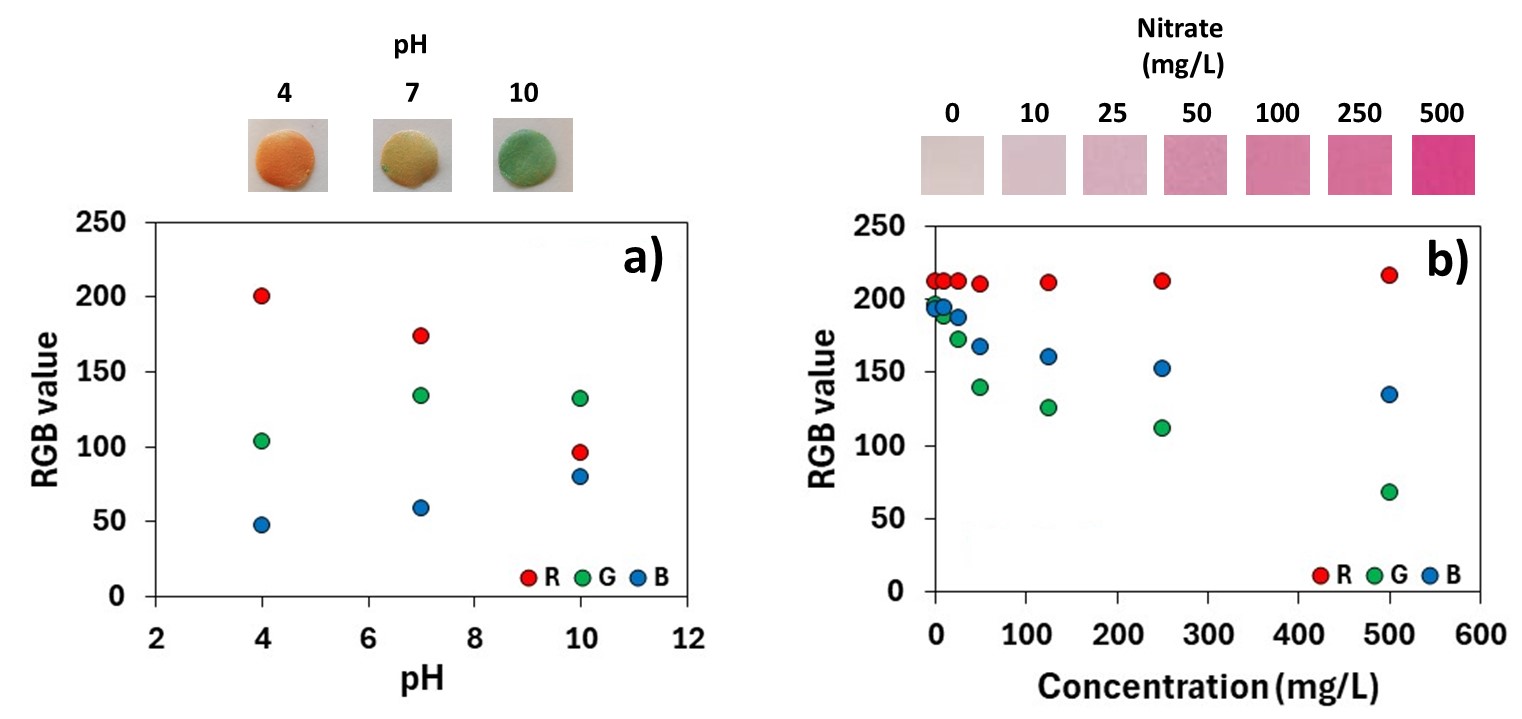
**

**Figure S11. Colorimetric analysis of pH and nitrate.** a) RGB values of colorimetric pH porous sensors at pH 4, 7 and 10. b) RGB values of the colorimetric nitrate sensors accordingly to the colour reference reported on the label at different nitrate concentrations (0-500 mg/L). RGB values were analysed using ImageJ.^[1]^

^
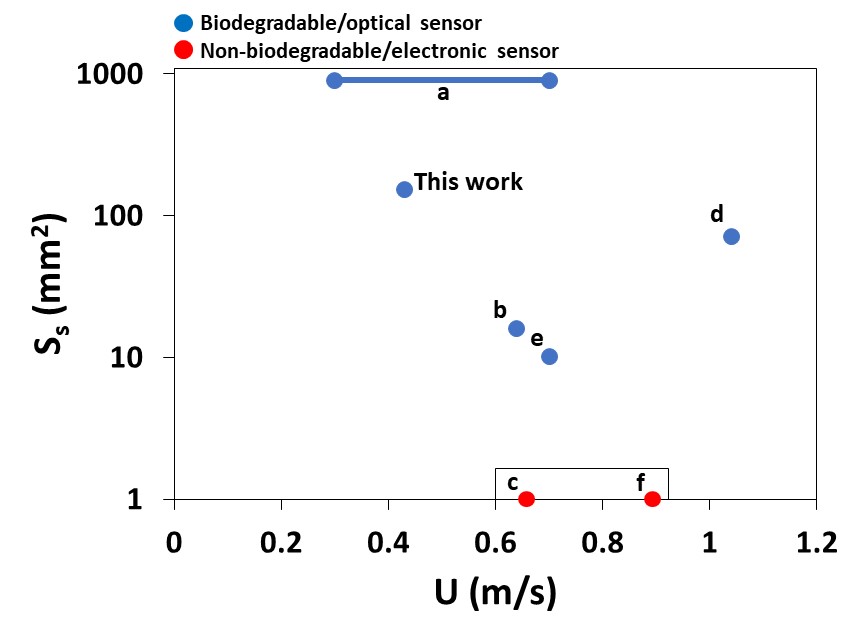
^

**Figure S12**. **State-of-the-art of seed-inspired fliers integrated with sensors for environmental monitoring.** For each integrated system is reported the descent speed (U, m/s) vs the sensor surface readable by drone camera (S_s_, mm^2^).

In the diagram are reported artificial fliers integrated with sensors and bioinspired to: a) *Alsomitra* (gliding);^5^ b) *Ailanthus*(autorotating*)*;^2^ c, d) *Acer* (autorotating);^6,7^ e) *Tristellateia* (autorotating);^8^ f and this work) *Tragopogon* (parachuting).^3^

Blue bullets report biodegradable fliers integrated with optical sensors, while red bullets report non-biodegradable fliers integrated with electronic sensors. In the case of the *Alsomitra* (a) is reported the descent speed of the natural specie. For electronic sensors, the surface is irrelevant for optical readings from the drone's camera. Therefore, we arbitrarily set it to 1 in the diagram. Data were taken from the reference manuscripts or extrapolated from the figures using ImageJ.^1^

This work reports the first flier bioinspired to a parachute flying fruit (i.e., *Tragopogon pratensis*), made with biodegradable materials and integrated with colorimetric sensor. The flier has one of the lowest descent speeds (U= 0.46 m/s) and one of the largest sensor surfaces (S_s_= 150 mm^2^). The combination of both enables a greater dispersal under wind conditions coupled with greater readability by the drone camera for perspective environmental monitoring.

**References**

1. C. A. Schneider, W. S. Rasband, K. W. Eliceiri, Nat Meth 2012, 9, 671.
2. K. Cikalleshi, S. Mariani, B. Mazzolai, in (Eds: F. Meder, A. Hunt, L. Margheri, A. Mura, B. Mazzolai), Springer Nature Switzerland, Cham, **2023**, pp. 117–129.
3. V. Iyer, H. Gaensbauer, T. L. Daniel, S. Gollakota, *Nature* **2022**, *603*, 427.
4. G. Li, H. Su, N. Ma, G. Zheng, U. Kuhn, M. Li, T. Klimach, U. Pöschl, Y. Cheng, Atmos. Meas. Tech. 2020, 13, 6053.
5. F. Wiesemüller, Z. Meng, Y. Hu, A. Farinha, Y. Govdeli, P. H. Nguyen, G. Nyström, M. Kovač, *Front. Rob*ot. AI **2022**, 9.
6. K. Cikalleshi, A. Nexha, T. Kister, M. Ronzan, A. Mondini, S. Mariani, T. Kraus, B. Mazzolai, Sci. Adv. 2023, 9, eadi8492
7. P. Pounds, S. Singh, *IEEE Potentials* **2015**, *34*, 10.
8. H.-J. Yoon, G. Lee, J.-T. Kim, J.-Y. Yoo, H. Luan, S. Cheng, S. Kang, H. L. T. Huynh, H. Kim, J. Park, J. Kim, S. S. Kwak, H. Ryu, J. Kim, Y. S. Choi, H.-Y. Ahn, J. Choi, S. Oh, Y. H. Jung, M. Park, W. Bai, Y. Huang, L. P. Chamorro, Y. Park, J. A. Rogers, Sci. Adv. 2022. 8, eade3201
